# Dynamic Mode Decomposition (DMD) for Low‐Latency Real‐Time Cardiac MRI

**DOI:** 10.1002/mrm.70360

**Published:** 2026-03-30

**Authors:** Ecrin Yagiz, Bilal Tasdelen, Ibrahim K. Ozaslan, Mihailo R. Jovanovic, Ye Tian, Krishna S. Nayak

**Affiliations:** ^1^ Ming Hsieh Department of Electrical and Computer Engineering, Viterbi School of Engineering University of Southern California Los Angeles California USA

**Keywords:** cardiac MRI, dynamic mode decomposition, image reconstruction, interactive imaging, online reconstruction

## Abstract

**Purpose:**

To demonstrate dynamic mode decomposition (*DMD*) for high spatiotemporal low‐latency online reconstruction in 2D real‐time cardiac MRI.

**Methods:**

DMD was applied to 2D spiral balanced steady state free precession (*bSSFP*) real‐time adult and fetal cardiac MRI at 0.55 T, with data from 10 healthy adult volunteers (3F/7M; age: 21–49; BMI: 20–34) and 6 pregnant females (maternal age: 30–41; maternal BMI: 22–47; gestational age: 23 weeks 6 days–37 weeks 5 days). DMD model appropriateness was assessed against off‐line spatiotemporally constrained reconstruction (*STCR*) as the reference. We retrospectively evaluated DMD‐based low‐latency online reconstruction at two temporal resolutions (21 and 42 ms/frame). DMD modes were estimated from the most recently acquired frames and used to remove aliasing while preserving underlying physiological motion.

**Results:**

DMD represented cardiac dynamics with normalized root‐mean‐square error (*NRMSE*) less than 7% when all modes retained. Low‐latency DMD‐based online reconstruction performed de‐aliasing while preserving the physiological motion, supporting framerates (21 and 42 ms/frame).

**Conclusion:**

We have demonstrated that the DMD framework is applicable to 2D real‐time cardiac MRI and for low‐latency de‐aliasing for better online reconstruction.

## 1 Introduction

Cardiac MRI is a powerful imaging tool capable of evaluating many aspects of the healthy and diseased heart, including regional wall motion, blood flow/hemodynamics, and myocardial perfusion, viability, microstructure, and other tissue properties [1]. Cardiac MRI requires respiratory and cardiac motion management, often in the form of breath‐holds, respiratory navigation, and cardiac gating via physiological monitoring of electrocardiogram (*ECG*) or photoplethysmography (*PG*) signals. Ventricular function imaging is a central part of almost every cardiac MRI scan [2], typically performed during a single breath‐hold per slice with ECG gating. CINE imaging is the noninvasive gold standard for the assessment of left‐ventricular (*LV*) anatomy, function, wall motion, and regional wall thickening [2]. However, there are some limitations to this approach. Cardiac gating may fail in patients with arrhythmia [3], and some patient cohorts may fail to comply with breath‐hold instructions, such as children or the incapacitated [4]. ECG gating also introduces additional complexity and cost (e.g., disposable leads). In the case of fetal cardiac MRI, ECG is replaced by Doppler ultrasound gating; however, the cardiac signal is not always reliable [5, 6]. This has led to substantial research toward contactless (e.g., pilot tone [7, 8, 9]) and data‐driven (e.g., self‐gating [10, 11, 12]) approaches for cardiac and respiratory monitoring.

Real‐time (*RT‐MRI*), on the other hand, continuously acquires data without relying on gating, synchronization, or the repetition of the underlying movement [13, 14]. This makes it possible to visualize dynamic processes with minimal assumptions, making it an appealing strategy for diagnostic applications that involve irregular movement, such as musculoskeletal, upper airway, gastrointestinal, and pediatric imaging [14, 15, 16, 17, 18, 19]. Specifically, in the context of ventricular functional imaging, RT‐MRI allows imaging in the setting of arrhythmia [3].

State‐of‐the‐art RT‐MRI methods with the finest spatiotemporal resolution typically utilize highly undersampled non‐Cartesian trajectories combined with a retrospective (and computationally intense) reconstruction that mitigates aliasing through a combination of parallel imaging, compressed sensing, and/or deep learning. To ensure proper localization, monitor subject motion, and/or guide interventions, however, real‐time interactive imaging (real‐time acquisition with low‐latency online reconstruction) is needed [13, 20]. Several online reconstruction approaches have been proposed in the literature. These approaches vary from simple techniques like view sharing [21, 22] to k‐t based methods [23], Kalman filter [24], constrained reconstruction [25, 26, 27], and deep learning [28, 29, 30, 31, 32, 33, 34]. View‐shared reconstructions are the fastest, but do not support high acceleration factors or fine temporal resolution. Deep learning approaches provide fast online reconstruction and support higher acceleration factors; however, they require an extensive training dataset, which is challenging to obtain in the context of RT‐MRI; hence, often synthetic datasets and CINE acquisitions are used as a proxy, limiting generalizability.

In this work, we propose a low‐latency online reconstruction routine for cardiac MRI using dynamic mode decomposition (*DMD*). DMD employs a system matrix representation of the underlying dynamics and extracts physically interpretable spatiotemporal features. Outside of MRI, DMD has proven valuable for applications including decomposing complex flow fields, weather analysis, computational neuroscience, and medical imaging [35, 36, 37, 38]. In the context of MRI, it has proven valuable for the separation of signal modulations caused by resting‐state functional connectivity in functional MRI [39] and ventilation and perfusion in functional lung imaging [40, 41]. We first evaluate the DMD model appropriateness on cardiac RT‐MRI data and then evaluate low‐latency online reconstruction for cardiac imaging. The proposed approach is independent of magnetic field strength. In this study, it is evaluated in adult and fetal cardiac RT‐MRI at 0.55 T.

## 2 Theory

### 2.1 DMD

DMD is a data‐driven method for dimensionality reduction and analysis that relies on measurements $x_{k}\in {\mathbb{C}}^{n}$ (called snapshots) collected from a dynamic system x(t), where $x_{k}=x\left(k\Delta t\right);k=1,\ldots ,m$ it is uniformly sampled in time with an interval Δt, n is the number of samples, and m is the number of measurements. In the context of dynamic MRI specifically, $x_{k}$ denotes the vectorized image at time frame k, where n corresponds to the number of pixels. DMD assumes the consecutive discrete measurements (time frames) are related by $x_{k+1}=Ax_{k}$ where $A\in {\mathbb{C}}^{n\times n}$ is a low‐rank approximation matrix that minimizes the error ${\left\|x_{k+1}-A\;x_{k}\right\|}_{2}$ across all m measurements [42, 43]. One can rearrange the measurements into two large matrices X and $X^{\prime }\in$ ${\mathbb{C}}^{n\times m-1}$,

(1) $$X=\left[\begin{array}{cc} | & |\\ x_{1} & x_{2}\\ | & | \end{array}\;\ldots \begin{array}{l} |\\ \;x_{m-2}\\ | \end{array}\begin{array}{l} |\\ \;x_{m-1}\\ | \end{array}\right]$$

$$X^{\prime }=\left[\begin{array}{cc} | & |\\ x_{2} & x_{3}\\ | & | \end{array}\;\ldots \begin{array}{l} |\\ x_{m-1}\\ | \end{array}\begin{array}{l} |\\ \;x_{m}\\ | \end{array}\right]$$

where X includes measurements from k=1 to m−1 and $X^{\prime }$ is shifted‐by‐one with measurements from k=2 to m. The matrix A can be defined as $A=X^{\prime }X^{+}$, where ${\left(.\right)}^{+}$ is the Moore–Penrose pseudoinverse. This definition corresponds to the linear operator that minimizes ${\left\|X^{\prime }-\mathrm{AX}\right\|}_{F}$, with ${\left\|.\right\|}_{F}$ the Frobenius norm [43, 44]. Given the input data pair $\left(X,X^{\prime }\right)$, DMD performs eigendecomposition on the state matrix A of the linear dynamical system that describes the transition from X to $X^{\prime }$, and it outputs the eigenvalues and eigenvectors (so‐called DMD modes) of A.

In practice, direct computation of the eigendecomposition may not be feasible; even storing the matrix *A* in memory can be prohibitive, especially with large dimensions *n* (number of pixels in a frame). For example, for an image of size n= 256 × 256, matrix A will occupy 32 GB of memory for complex‐valued single precision data. The DMD algorithm calculates the eigen decomposition without explicitly calculating the matrix A by considering a rank‐reduced representation in terms of a projected matrix $A_{r}$. First, the rank‐r approximation of X is calculated via SVD truncation, $X_{r}=U_{r}\Sigma_{r}V_{r}^{*}$, with ${\left(.\right)}^{*}$ conjugate transpose, $U_{r}\in {\mathbb{C}}^{n\times r}$, $\Sigma_{r}\in {\mathbb{C}}^{r\times r}$, and $V_{r}\in {\mathbb{C}}^{\left(m-1\right)\times r}$ are rank‐reduced SVD components of $X=U\Sigma V^{*}$. Then, $A_{r}$ is defined as

(2) $$\begin{array}{c} A_{r}≜U_{r}^{*}A\;U_{r}=U_{r}^{*}X^{\prime }X^{+}U_{r}=U_{r}^{*}X^{\prime }V_{r}\;\Sigma_{r}^{-1} \end{array}$$

The eigen decomposition is carried out over $A_{r}\in {\mathbb{C}}^{r\times r}$, with $A_{r}W=W\Lambda$, where the columns of W are the eigenvectors and Λ is a diagonal matrix of corresponding eigenvalues. Then, the eigendecomposition of the original matrix A can be calculated using this rank‐reduced computation. The eigenvalues of $A_{r}$ correspond to those of A with the highest energy in terms of the Frobenius norm given above. Moreover, the dynamic modes (eigenvectors) of A can be recovered as [44]

(3) $$\begin{array}{c} \Phi =X^{\prime }V\;\Sigma^{-1}W \end{array}$$

The dynamic modes (ϕ) show coherent spatial correlations, and eigenvalues (λ) show temporal characteristics of the corresponding mode, that is, growth/decay and oscillations (temporal frequency). The temporal frequency of a mode is calculated from its eigenvalue (λ) by f=lnλΔt [43, 44]. Finally, one can construct individual snapshots $\left(x_{k}\right)$ or predict future time steps from the DMD components via

(4) $$\begin{array}{c} x_{k}\approx \sum_{l=1}^{L}b_{l}\phi_{l}\lambda_{l}^{k} \end{array}$$

where b is a vector of coefficients, which can be chosen using the initial condition, and $k\in {\mathbb{Z}}^{0+}$ is the frame index. Please refer to Tu et al. [44] for more details.

## 3 Methods

### 3.1 Experimental Methods

All experiments were performed on a whole‐body 0.55 T system (prototype MAGNETOM Aera, Siemens Healthineers, Forchheim, Germany) equipped with high‐performance gradients (45 mT/m amplitude, 200 T/m/s slew rate) [45]. The body coil was used for RF transmission. For adult cardiac imaging, a 6‐channel surface body coil (anterior) and 6 elements from an 18‐channel spine array (posterior) were used for signal reception. For fetal cardiac imaging, 6–12 elements from the spine array (posterior) and 1 or 2 body‐6 array (anterior) were used, depending on subject positioning [46, 47]. Data were collected from 10 adults (3F/7M; age 29.8 ± 8.7; BMI 25.7 ± 4) and 6 fetal (maternal age 34.5 ± 3.7, gestational age 31 weeks 6 days ± 4 weeks, maternal BMI 29.5 ± 9.7). All subjects were scanned under protocols approved by our Institutional Review Board (IRB HS‐21‐00250 and HS‐22‐00534), after providing written informed consent.

Pulse sequences were implemented using the RTHawk system [48] (Vista.AI Inc., Menlo Park, CA) and PyPulseq [49, 50]. Imaging was performed with 2D real‐time bSSFP pulse sequences with golden‐angle spiral readouts. The readouts were uniform‐density spiral trajectories with a duration of 3 ms with an additional M1‐nulled rewinder for adults [51]. All reconstructions used gradient impulse response function (*GIRF*) corrected trajectories [52]. Scan parameters for adult cardiac imaging: spatial resolution = 2.2 × 2.2 mm^2^, slice thickness = 8 mm, TE/TR = 0.72/5.3 ms. The flip angle (FA) was set to 100°, based on prior experimental optimization of blood‐myocardium contrast [53, 54]. Scan parameters for fetal cardiac imaging: the spatial resolution was set to one of the following: 1.5 × 1.5, 1.7 × 1.7, 2.2 × 2.2 mm^2^ based on the perceived image quality during the scan [47], with slice thickness = 4 mm, TE/TR = 0.72–0.89/5.28–5.72 ms, and FA was set to 90°– 120°, based on prior experimental optimization of blood‐myocardium contrast [47].

The proposed low‐latency approach is validated against an offline spatiotemporally constrained reconstruction (*STCR*) [55]. This scheme was chosen because various real‐time imaging applications have demonstrated its use [47, 54, 55, 56, 57]. STCR minimizes the following cost function using a nonlinear conjugate gradient algorithm with a line search:

(5) $$\begin{array}{c} {\left\|\mathrm{Am}-d\right\|}_{2}^{2}+\lambda_{s}{\left\|\sqrt{{\left(\nabla_{x}m\right)}^{2}+{\left(\nabla_{y}m\right)}^{2}+\epsilon }\right\|}_{1}+\lambda_{t}{\left\|\sqrt{{\left(\nabla_{t}m\right)}^{2}+\epsilon }\right\|}_{1} \end{array}$$

where d is the multicoil k‐space data, A=FS is the encoding matrix, F is the nonuniform Fourier transform, and S represents coil sensitivities that are estimated by the Walsh method [58]. m represents the image series to be reconstructed, and ϵ is a small positive value to avoid singularity issues. $\lambda_{t,s}$are temporal and spatial regularization parameters, which were optimized in previous studies and set to 0.05/0.005 for adult cohort [54] and 0.02/0.002 for fetal [47], respectively.

### 3.2 Model Appropriateness

To study DMD model appropriateness in the context of cardiac RT‐MRI, we investigated DMD model residue energy as a function of the number of modes. We applied DMD to the STCR reconstructed image series $x_{k}^{\text{STCR}}$, $k\in \left[1,N_{\text{frames}}\right]$. Then, a subset of modes l (from L= 1 to $L=N_{\text{frames}}$‐1) was selected. These modes were composed back using the Sparsity‐Promoting Dynamic Mode Decomposition (*DMDSP*) algorithm [59] to reconstruct an image series. The resulting reconstruction, $x_{l,k}^{\mathrm{DMD}}$, represents the model capacity and was computed using Equation (4), where the coefficient vector b was obtained from DMDSP. The model residue ($r_{l,k}^{\text{STCR}}$) was identified as the difference between the original input ($x_{k}^{\text{STCR}}$) and the model estimation using l modes ($r_{l,k}^{\text{STCR}}=x_{k}^{\text{STCR}}-x_{l,k}^{\mathrm{DMD}}$).

The normalized energy of the residue was calculated as ${\left\|r_{l,k}^{\text{STCR}}\right\|}_{2}/{\left\|x_{k}^{\text{STCR}}\right\|}_{2}$. This procedure was repeated for increasing number of modes (l) until the maximum number of modes was reached.

### 3.3 Low‐Latency Real‐Time Cardiac MRI

We hypothesized that DMD can be utilized for a low‐latency, high‐temporal resolution RT‐MRI by representing the dynamics associated with the undersampling artifact. The sampling schemes for RT‐MRI (in this study spiral golden‐angle) change as a function of time (frames). Hence, when undersampled, the aliasing artifact is expected to be captured in dynamic modes. The golden‐angle interleaf order is widely used in RT‐MRI, and the temporal modulations are expected to occur at frequency bands that differ from the physiologically relevant motion such as respiratory or cardiac motion. Furthermore, the number of fundamental harmonics associated with relevant motions is limited, allowing separation of the aliasing and tissue signal (de‐aliasing) based on a frequency‐based threshold.

**FIGURE 1:**
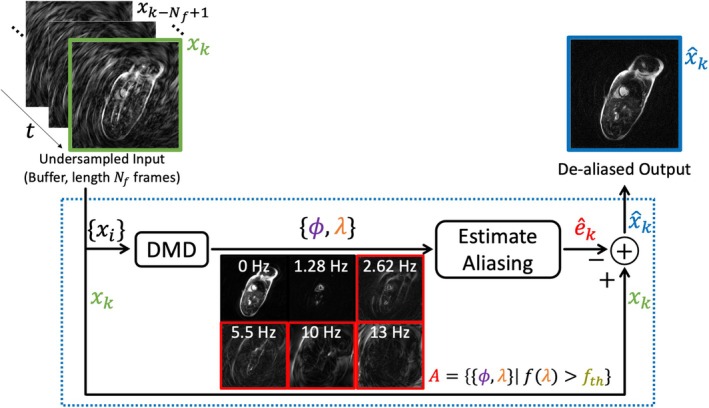
Flowchart of the proposed low‐latency reconstruction using dynamic mode decomposition (DMD). First, every nonoverlapping N_arms_ read‐out (spiral arm) is grouped and gridded to construct an undersampled frame $x_{i}$. The inputs to the low‐latency reconstruction are the most recent $N_{f}$ frames (buffer: ${\left\{x_{i}\right\}}_{k-N_{f}+1}^{k}$) and the frequency threshold $\;f_{\mathrm{th}}$. All $N_{f}$ frames are used in decomposition; some example modes are shown. Then, a set of aliasing modes (A), some shown marked with red, is identified based on the frequency of the modes. Aliasing for the current frame (${\hat{e}}_{k}$) is estimated. Finally, the output (${\hat{x}}_{k}$) is a single frame computed as the difference of the current frame ($x_{k}$) and estimated aliasing (${\hat{e}}_{k}$).

Figure 1 shows the flowchart of the proposed approach. First, the nonoverlapping sets of $N_{\text{arms}}$ (set to 4 or 8 in this study corresponding to 21 and 42 ms temporal resolution) incoming readouts (spiral arms) are grouped and gridded to form an undersampled frame $x_{i}$. Then, the input to the low‐latency reconstruction is the last $N_{f}$ gridded 2D complex‐valued real‐time frames in the current buffer. DMD is applied to the input image series in the buffer. Based on the input threshold frequency $\left(f_{\mathrm{th}}\right)$, aliasing dynamic modes and their temporal evolutions $\left\{\phi_{A},\lambda_{A}\right\}$ are selected. Aliasing artifact $\left({\hat{e}}_{k}\right)$ is then estimated by,

(6) $$\begin{array}{c} {\hat{e}}_{k}=\sum_{l\in A}b_{l}\phi_{l}\lambda_{l}^{k} \end{array}$$

where A denotes the set of indices of components selected as aliasing modes, with frequency higher than $f_{\mathrm{th}}$. Finally, a dealiased output image series $\left({\hat{x}}_{k}\right)$ is produced by subtracting the aliasing artifact estimate image ${\hat{e}}_{k}$ from the input image,

(7) $$\begin{array}{c} {\hat{x}}_{k}=x_{k}-{\hat{e}}_{k} \end{array}$$

Once the current frame ($x_{k}$) is de‐aliased, the buffer moves by one frame. In this study, we used $f_{\mathrm{th}}$ of 2 Hz for adult and 3 Hz for fetal imaging to preserve the first harmonics of the cardiac motion. These values are based on the cardiac self‐gating literature [60] and a slightly higher one to accommodate fetal heartbeat [61].

We investigated the de‐aliasing performance as a function of the buffer length ($N_{f}\times \text{temporal resolution}$) at two temporal resolution (21/frame and 42 ms/frame corresponding to 4 and 8 TR/frame) that is used in real‐time imaging [14]. On adult and fetal datasets acquired with the outlined acquisition scheme, we performed gridding. We applied the proposed low‐latency reconstruction using buffer length of 85 ms to 2.1 s (2–50 frames at 8 TR/frame and 4–100 frames at 4 TR/frame). Five quantitative assessments were performed: normalized mean‐square error (*NMSE*), learned perceptual image patch similarity (*LPIPS*) [62], high‐frequency error norm (*HFEN*) [63], peak signal‐to‐noise ratio (*PSNR*), and structural similarity index measure (*SSIM*) [64]. We evaluated selected metrics between low‐latency reconstructed images (gridding + DMD based de‐aliasing) and the reference offline STCR reconstructed images at the same temporal resolution.

For the fetal cardiac RT‐MRI de‐aliasing, we introduced an additional parameter, residue scale factor α∈[0,1] to further suppress the aliasing using the residual signal ($r_{k}$) from the undersampled image series. The residual signal ($r_{k}$) here is defined as the difference between the gridded image series ($x_{k}$) and the DMD model estimation with all available modes ($r_{k}=x_{k}-\sum_{l=0}^{N_{f}-1}b_{l}\phi_{l}\lambda_{l}^{k}$). Specifically, for fetal cardiac RT‐MRI, Equation (7) is modified as,

(8) $$\begin{array}{c} {\hat{x}}_{k}=x_{k}-{\hat{e}}_{k}-\alpha \;r_{k} \end{array}$$

We investigated the effect of residue scale factor α∈[0,1] qualitatively on the fetal dataset at both temporal resolutions. We applied the DMD based low‐latency method with the experimentally established optimum buffer length $N_{f}$ and swept α values. All results were evaluated qualitatively via assessing the performance of dealiasing in terms of the fetal heart visibility for interactive localization and the fidelity of the reconstruction (i.e., truthfulness of the underlying body motion with respect to the offline STCR reference).

Then, we evaluated the run‐time performance as a function of image size (n) and the buffer length $\left(N_{f}\right)$. Testing was performed on a server with 4× AMD EPYC 7502 32‐core CPUs and 4× NVIDIA A100 GPUs (40GB memory). The proposed approach was implemented for GPU using the CuPy library [65]. The implementation used CPU for the eigenvalue decomposition of the projected matrix $\tilde{A}\in {\mathbb{C}}^{r\times r}$ and a single GPU core for the rest of the operations. Benchmarking was performed using CuPy's profiler routine.

Reconstructed images were qualitatively evaluated using offline STCR reference and gridded images on all datasets at both selected temporal resolutions. This qualitative analysis was performed on all data and focused on the fidelity of the reconstruction of the heart and its motion.

## 4 Results

### 4.1 Appropriateness of DMD in Cardiac MRI

**FIGURE 2:**
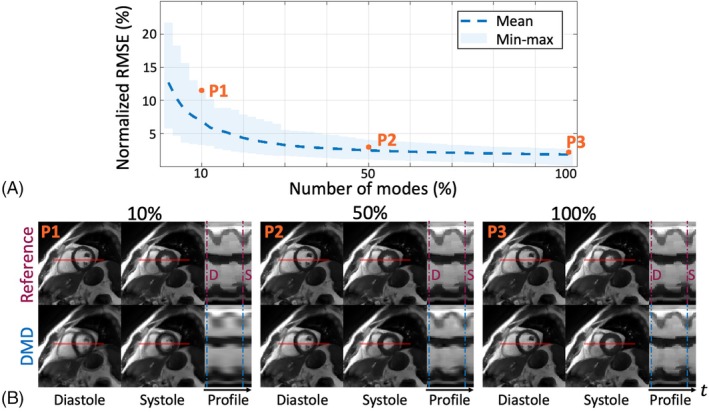
Evaluation of DMD model appropriateness for 2D cardiac RT‐MRI. DMD was applied to the spatiotemporally constrained reconstruction (*STCR*) on all adult datasets. Then, a varying number of dynamic modes (shown as percentage of maximum available modes) were used to generate a DMD reconstructed image series. The model appropriateness is defined as the root‐mean‐square error (*RMSE*) normalized by the input energy, given a DMD reconstruction. (A) Normalized RMSE of model error with respect to the number of modes on all adult datasets. Note that as the number of modes increases, the normalized RMSE decreases; however, it is always nonzero as the DMD is not an orthogonal transformation. (B) A representative dataset (M; 49 years old; BMI 29) with one of the highest normalized RMSE (P1, P2, and P3 are examples showing model capacity at 10%, 50% and 100% number of modes). The DMD representations at 50% and 100%, qualitatively represent the dynamics faithfully. Video S1 shows the corresponding movie. Video S2 shows the same analysis for the fetal dataset.

We show the DMD model capacity in terms of the normalized RMSE with respect to the number of modes in Figure 2A, where DMD was applied to the STCR reconstructed images. Normalized RMSE was calculated with offline STCR reconstruction as the reference, to study model appropriateness. Normalized RMSE decreases as the number of modes increases; however, it is always nonzero as the DMD is not an orthogonal transformation. Figure 2B shows an example from the adult dataset (M, 49 years BMI 29.1) with one of the highest normalized RMSE error and Video S1 shows the corresponding movie. The dynamics are captured as can be seen from the DMD representation. Video S2 shows a similar analysis for the fetal dataset together with an example representation. Similarly, the normalized RMSE decreases as the number of modes increases and the original input dynamics are captured including other fetal motion shown with arrows.

### 4.2 Low‐Latency Real‐Time Cardiac MRI

**FIGURE 3:**
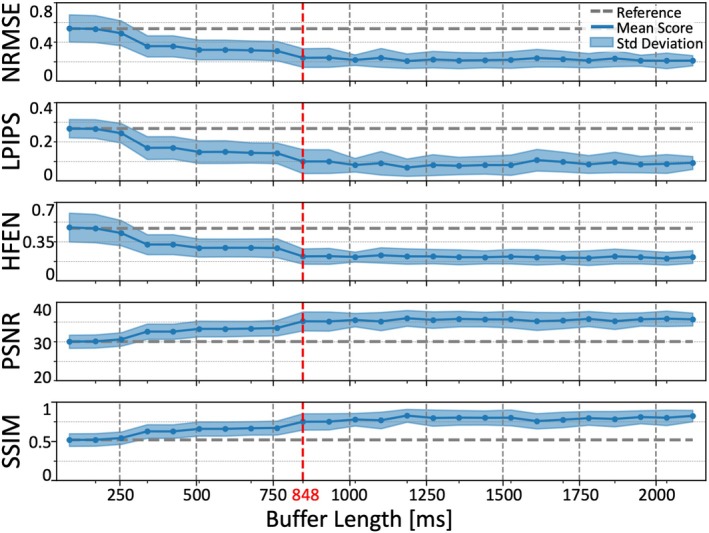
Impact of DMD buffer length on image quality metrics. We performed gridding on all adult datasets. Then, retrospectively applied the proposed low‐latency reconstruction using different buffer lengths. De‐aliasing performance was assessed using STCR reconstructions as a reference by normalized RMSE (*NRMSE*), high‐frequency error norm (*HFEN*), learned perceptual image patch similarity (*LPIPS*), peak signal‐to‐noise ratio (*PSNR*), and structural similarity index measure (*SSIM*). We selected the buffer length of 848 ms ($N_{f}=20$ at 8 TR/frame; $N_{f}=40$ at 4 TR/frame), since larger buffers increased computation time without meaningful metric improvement. Figure S1 shows the same analysis for the fetal dataset where the same buffer length was selected.

The proposed DMD‐based method was applied to the gridded images to investigate the image quality for a low‐latency online reconstruction and was compared against the offline STCR reconstructed images. Figure 3 shows the de‐aliasing performance as a function of the buffer length ($N_{f}$). Plots show the NRMSE, LPIPS, HFEN, PSNR and SSIM metrics between the proposed approach and the STCR reference at increasing buffer lengths. We selected the buffer length of 848 ms, since larger buffers increased computation time without significant metric improvement. The buffer length of 848 ms corresponds to 20 frames at the 8 TR/frame (42 ms/frame) temporal resolution and 40 frames at 4 TR/frame (21 ms/frame). Figure S1 shows the same analysis for the fetal dataset, similarly 848 ms is selected as the optimal buffer length given the image quality metrics. Note that the length of the buffer is not the temporal resolution of the proposed routine, it is the temporal footprint at which decomposition is applied to capture the input dynamics.

**FIGURE 4:**
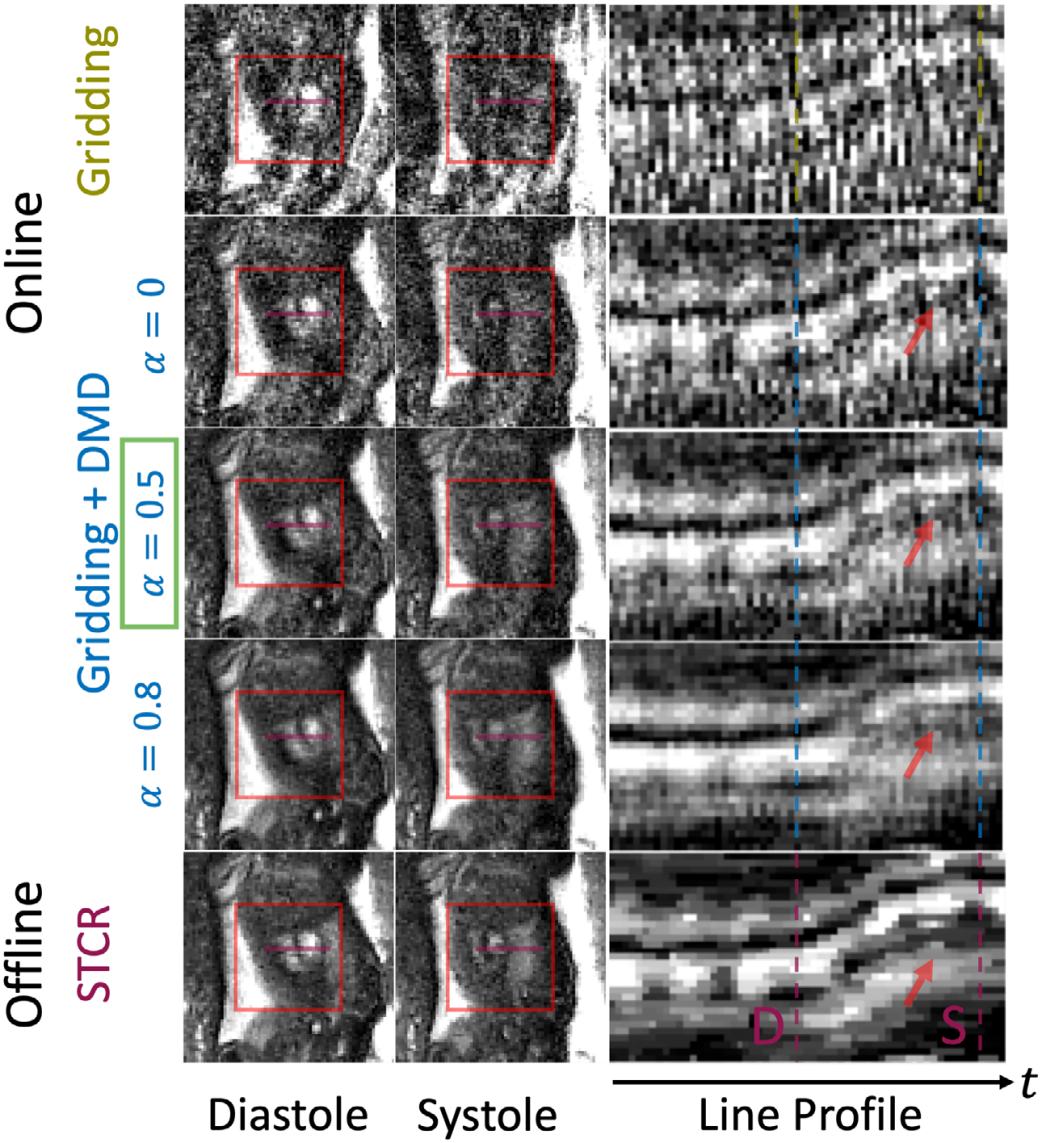
Impact of residue scale factor (α) on de‐aliasing performance for fetal cardiac RT‐MRI. The difference between the gridded input image series and the DMD model estimation, that is, residual signal, can be used to further suppress the aliasing. Specifically, the residual signal is scaled with parameter α∈[0,1] (residue scale factor) and added to the estimated aliasing with little computational overhead (no iterations). The effect of α is shown here for 0, 0.5 and 0.8 on an example fetal dataset with significant bulk motion (marked in the line profile), Video S3 shows the corresponding movie. Larger α values support better dealiasing performance at the cost of temporal smoothing, while smaller α values preserve the dynamics better at the cost of increased aliasing. We selected α=0.5 after a qualitative analysis on the fetal dataset as it provides a balance between the de‐aliasing performance and preservation of the dynamics.

Figure 4 shows the residue scale factor α sweep for fetal cardiac RT‐MRI and Video S3 shows the corresponding movie. The de‐aliasing performance and the dynamics are shown from a healthy pregnancy (gestational age 32 weeks 2 days, maternal BMI 24) with bulk fetal motion (can be seen in line intensity profile and Video S3). The residue scale factor α acts as a temporal regularization. Larger α values support better dealiasing performance at the cost of temporal smoothing, while smaller α values preserve the dynamics better at the cost of increased aliasing. We selected α=0.5 after a qualitative analysis on the fetal dataset as it provides a balance between the de‐aliasing performance and preservation of the dynamics.

**FIGURE 5:**
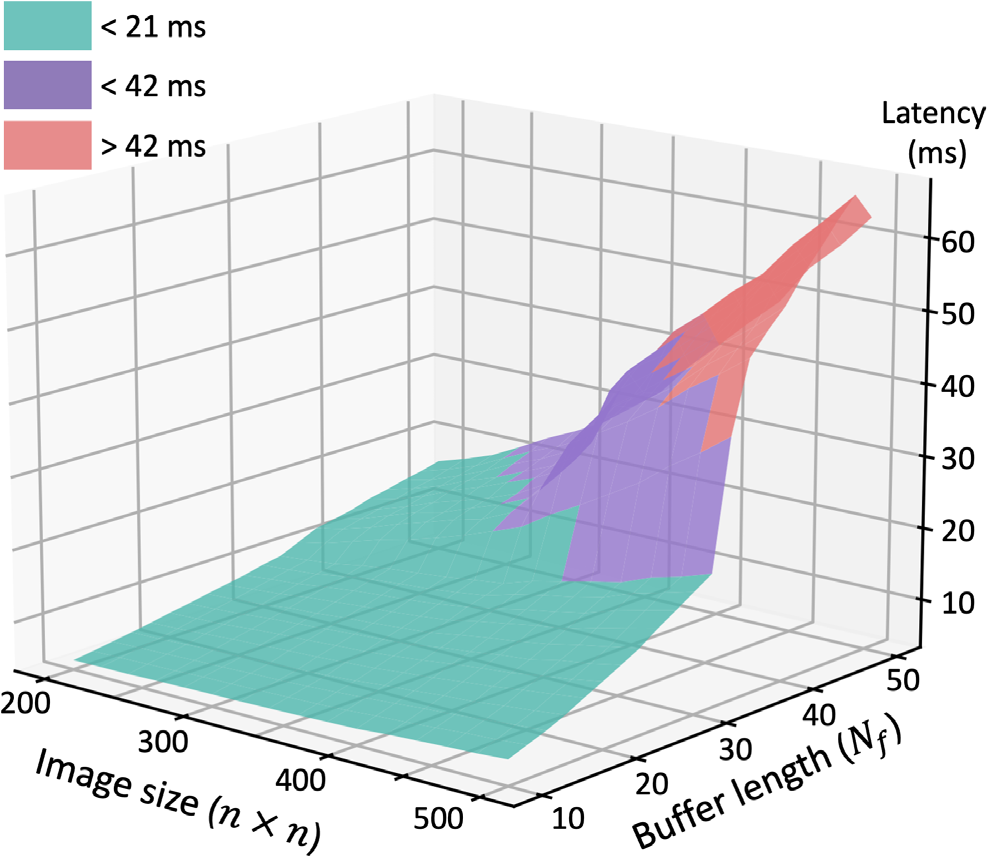
Run‐time performance of the proposed low‐latency reconstruction. For relevant image matrix sizes (200 × 200 to 500 × 500) and number of frames (buffer length of 10–50) we evaluated the run‐time of the proposed method. Testing was performed on a server equipped with 4× AMD EPYC 7502 32‐core CPUs and 4× NVIDIA A100 GPUs (40GB memory for each). Reconstruction used a single GPU. The elapsed run‐time was compared against two relevant framerates: 42 ms (eight spiral arms/frame) and 21 ms (four spiral arms/frame). For most of the relevant image sizes (using optimal buffer length from Figure 3), the method can catch up to the framerate.

Figure 5 shows the run‐time performance of the DMD‐based dealiasing alone with respect to image matrix size (n) and the buffer length $\left(N_{f}\right)$. The proposed approach can satisfy the latency requirement at the temporal resolutions of 21 ms (4 TR/frame) and 42 ms (8 TR/frame) for image sizes n = 500 × 500 using the corresponding optimal buffer lengths of $N_{f}$ = 40 and 20, respectively.

Table S1 shows the qualitative image metrics against the offline constrained reconstruction with the experimentally established optimal parameters for both cohorts at two temporal resolutions. Arrows indicate the preferred direction in a given metric (higher ↑/lower ↓). The same image quality metrics were obtained for gridded images as a baseline. The proposed DMD‐based approach shows improvement of the image quality providing low‐latency reconstruction.

**FIGURE 6:**
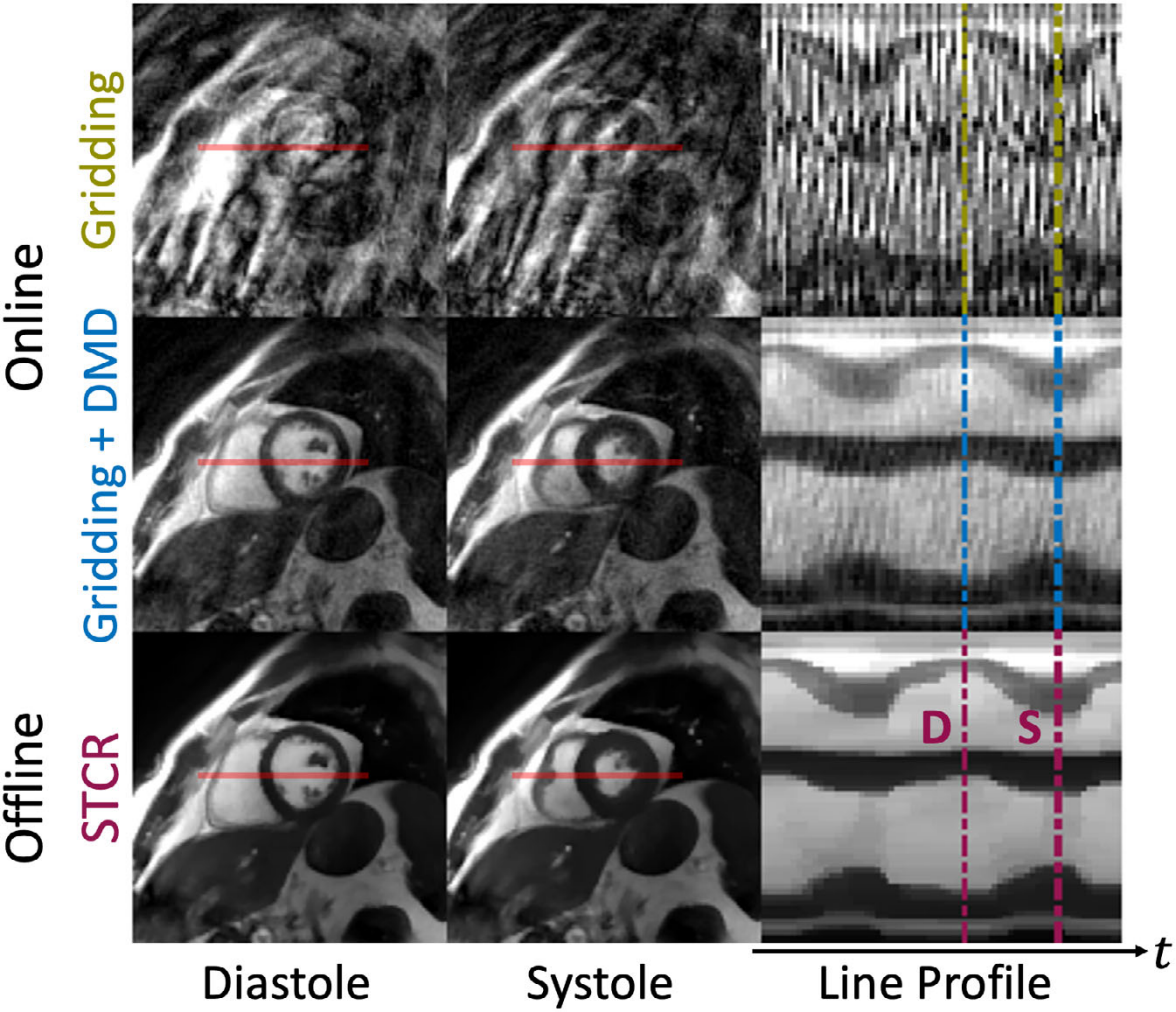
De‐aliasing performance of the proposed approach. Shown are end‐diastolic and end‐systolic frames at 21.2 ms (4 TR/frame) temporal resolution, from a healthy adult volunteer (M, 49 years old, BMI 29), the same volunteer from Figure 2. DMD‐based dealiasing is applied to the gridded image series; results are shown together with STCR results at the same temporal resolution. Video S4 shows the movie of the results at 21.2 ms (4 TR/frame) and 42.4 ms (8 TR/frame) temporal resolutions. This illustrates that the proposed approach, Gridding + DMD, can output de‐aliased images (compared to Gridding alone) with good fidelity while preserving underlying motion (compared to offline STCR).

The DMD based de‐aliasing shows improvement in the selected image quality metrics for both cohorts and temporal resolutions with respect to the offline STCR reference. Figure 6 shows an example low‐latency reconstruction (gridding + DMD) result, with gridding and STCR reconstructed images at end‐diastole and end‐systole frames at 21.2 ms temporal resolution (4 TR/frame) on the same volunteer in Figure 2. Video S4 shows the movie of the low‐latency reconstruction results at two temporal resolutions synchronized in the oversampled field of view. This illustrates that the proposed approach, Gridding + DMD, can output de‐aliased images (compared to gridding) with good fidelity while preserving underlying motion (compared to offline STCR).

Video S5 shows the de‐aliasing performance in the presence of irregular cardiac cycles in a healthy volunteer (male, age: 28, BMI: 26) experiencing premature ventricular contraction (*PVC*) at two temporal resolutions in the oversampled field of view. The line intensity profiles highlight the irregular beat. Low‐latency reconstruction provides de‐aliased results while preserving the irregular underlying motion, such as an elongated cardiac beat.

**FIGURE 7:**
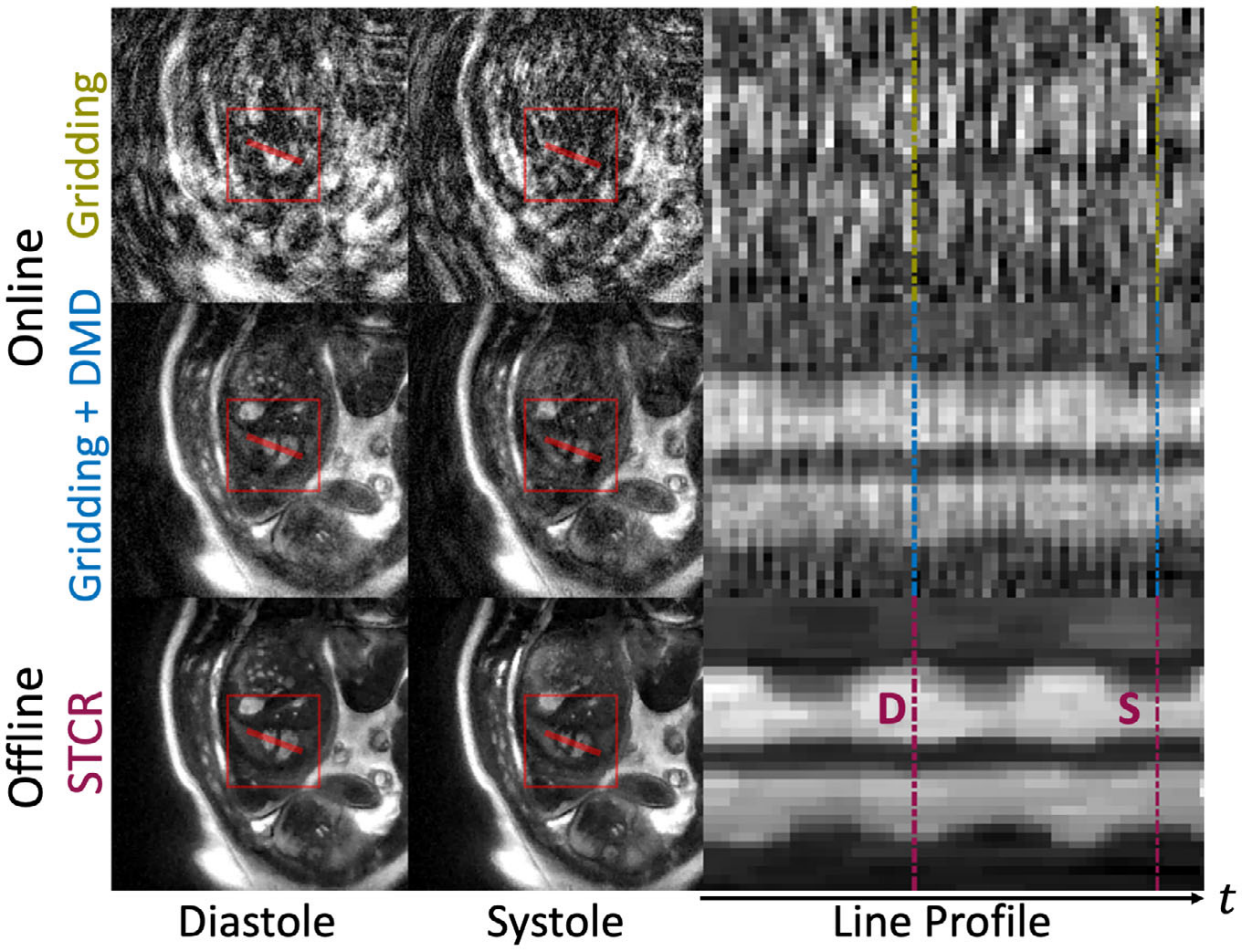
Feasibility of the proposed low‐latency de‐aliasing approach for fetal cardiac RT‐MRI. Shown are end‐diastolic and end‐systolic frames at 21.2 ms (4 TR/frame) temporal resolution. DMD‐based de‐aliasing is applied to the gridded image series; results are shown together with STCR results at the same temporal resolution. Video S7 shows the real‐time movie of the results of the same dataset at 21.2 ms (4 TR/frame) and 42.4 ms (8 TR/frame) temporal resolutions. The proposed reconstruction can perform rapid de‐aliasing to localize the fetal heart with adequate temporal resolution to guide localization.

Finally, we show the feasibility of using the proposed low‐latency reconstruction for fetal cardiac RT‐MRI through two healthy pregnancies. First, Figure 7 shows de‐aliasing performance at 4 TR/frames temporal resolution (same volunteer as Video S2) and Video S6 shows the corresponding movie at two temporal resolutions. The line intensity profiles show that the proposed low‐latency reconstruction can support high temporal resolution to resolve fetal heartbeats. Finally, Video S7 shows a very challenging imaging example with high maternal BMI of 47 (gestational age 31 weeks 4 days), a scenario where online de‐aliasing methods are useful. The fetal heart and the cardiac cycle can be identified in the DMD dealiased results even at finer spatial resolution (4 arms/frame).

## 5 Discussion

We have demonstrated that the DMD framework is applicable to 2D cardiac RT‐MRI. Furthermore, we demonstrate the feasibility of a specific use case of low‐latency de‐aliasing for better RT‐MRI online reconstruction which is demonstrated in the context of adult and fetal cardiac MRI at 0.55 T.

Although this work was performed at 0.55 T, the proposed methods are field strength agnostic. The 0.55 T field strength has the benefit of allowing for longer bSSFP TR, use of efficient readouts [66], and higher FAs [53], but has lower intrinsic equilibrium polarization. On the other hand, at conventional field strengths such as 1.5 T or 3 T, shorter bSSFP TR values are used to avoid banding artifacts. Therefore, it would be appropriate to use shorter spiral readouts or switch to radial or Cartesian readouts.

Low‐latency, high‐quality RT‐MRI reconstruction has many potential applications. Fetal cardiac localization, the example demonstrated in this work, is extremely challenging due to the high heart rate and small feature size. The fine spatial resolution requirement demands a long imaging window for a fully sampled image, typically longer than the fetal R‐R interval. This makes conventional view‐sharing reconstructions incapable of capturing cardiac dynamics [47]. The proposed method, if implemented on a real‐time system, would overcome this challenge. Additional applications include the guidance of interventions [20, 67] and biofeedback [68, 69].

Compared with deep learning‐based reconstruction methods, the proposed DMD approach does not require training data, large curated datasets, or re‐training to generalize across different acquisition schemes, patient populations, and so on. While deep learning methods can achieve excellent image quality, their performance may degrade under domain shift and often introduce higher computational complexity for low‐latency reconstruction.

DMD does not require any training data but has a few important manually defined parameters. The low‐latency reconstruction routine assumes a heartbeat smaller than a threshold frequency $\left(f_{\mathrm{th}}\right)$, which was selected as 2 and 3 Hz for adult and fetal cardiac, respectively, reflecting the average heartbeat frequency. These thresholds were chosen to accommodate heart‐rate variability. When applying DMD to new patient groups, it is appropriate to rethink these parameters. In addition, fetal de‐aliasing uses a user‐defined residual scaling factor (α), which acts as a temporal regularization parameter without requiring iterative optimization. This parameter may also need adjustment for different patients. Importantly, all parameters can be potentially updated during scanning, enabling online tuning based on the feedback from the reconstruction.

The low‐latency reconstruction was tested only with a 2D bSSFP golden‐angle spiral acquisition. We found that processing the incoming k‐space data stream in chunks of 848 ms (20 frames with 8 spiral arms per frame; 40 frames with 4 spiral arms per frame) qualitatively led to the best de‐aliasing performance. This means that the first de‐aliasing output is available < 1 s after the scan has started. This is not due to the latency of the reconstruction, but to accumulate data in the sliding window buffer. After this initialization, the sliding window buffer will always be full, and de‐aliasing of the current frame will be completed before the next frame arrives. The optimal length of the data stream may change with the acquisition trajectory. Overall, the de‐aliasing performance may change based on the acquisition scheme, undersampling factor, and the maximum heartbeat. Note that we have only focused on the de‐aliasing performance from a localization standpoint; other downstream tasks, such as volume calculations, need to be investigated further.

The proposed low‐latency de‐aliasing exploits the semi‐periodicity of cardiac and respiratory motions. The number of fundamental harmonics associated with these motions is limited. Hence, a good separation between the aliasing artifacts and the desired MR signal is possible. We do not expect DMD to perform well in applications like speech production or gastrointestinal motility, where this assumption does not hold.

This study has several limitations. First, the method was tested in a relatively small sample. While adequate to demonstrate feasibility, this is not sufficient to make claims about robustness or potential clinical application. This requires additional testing in larger, diverse, and clinically‐relevant cohorts (e.g., varying body composition, age, sex, underlying health conditions). Second, the method was only tested in the context of spiral bSSFP at 0.55 T. This was chosen as it is the best performing approach for RT‐MRI ventricular function assessment at this field strength. While we do not expect the method to fundamentally change with B0 field strength, it should be tested before making claims of generalizability. Third, DMD model appropriateness was evaluated in the context of adult and fetal cardiac imaging in a handful of cases. It is possible that the model appropriateness is reduced in extremely challenging cases (e.g., uncontrolled body motion, exercise stress, severe arrhythmia). This was not tested and requires further investigation. Note that it is challenging to evaluate model appropriateness in such cases because reference data may not exist.

## 6 Conclusion

We have shown the appropriateness of dynamic mode decomposition (DMD) for 2D real‐time cardiac MRI and demonstrated its feasibility for low‐latency de‐aliasing to improve online cardiac RT‐MRI reconstruction.

## Funding

This work was supported by the National Institutes of Health (R01‐HD115284, R21‐HL159533, U01‐HL167613).

## Conflicts of Interest

Our group receives research support from Siemens Healthineers.

## Supporting information


**Figure S1:** Impact of DMD buffer length on image quality metrics, for fetal cardiac RT‐MRI. We performed gridding on all fetal datasets. Then retrospectively, we applied the proposed low‐latency reconstruction using different buffer lengths. The performance of the de‐aliasing was assessed using spatiotemporally constrained reconstruction (STCR) as a reference by normalized RMSE (NRMSE), high‐frequency error norm (HFEN), learned perceptual image patch similarity (LPIPS), peak signal‐to‐noise ratio (PSNR), and structural similarity index measure (SSIM). We found the sliding window width of 848 ms ($N_{f}=$ 20 frames at 8 TR/frame and $N_{f}=$ 40 frames at 4 TR/frame) to be optimal.
**Table S1:** Summary of image quality metrics for the adult and fetal cohort at two temporal resolutions (4 and 8 TR/frame). The performance of the de‐aliasing was assessed using STCR reconstructions by normalized RMSE (NRMSE), high‐frequency error norm (HFEN), learned perceptual image patch similarity (LPIPS), peak signal‐to‐noise ratio (PSNR), and structural similarity index measure (SSIM). Arrows indicate the preferred direction in a given metric (higher ↑/lower ↓). The same image quality metrics were obtained for gridded images as a baseline. The proposed DMD‐based approach (gridding + DMD) shows improvement of the image quality providing low‐latency reconstruction (can provide framerates of 21 ms).

**Video S1:** Representative example of DMD model capacity for adult cardiac RT‐MRI. DMD was applied to the spatiotemporally constrained reconstruction (STCR) results. Then, a varying number of dynamic modes (shown as percentage of maximum available modes) were used to generate a DMD reconstructed image series. The model appropriateness is defined as the root‐mean‐square error (RMSE) normalized by the input energy, given a DMD reconstruction. Figure 2A shows the normalized RMSE of model error with respect to the number of modes on all adult datasets. Here, an example dataset (M; 49 years old; BMI 29) with one of the highest normalized RMSE is shown (movie of Figure 2B). As can be seen from the DMD representations at 50% and 100%, qualitatively DMD can represent the original input dynamics faithfully. Video S2 shows the same analysis for the fetal dataset.

**Video S2:** DMD model appropriateness for 2D fetal cardiac RT‐MRI. DMD was applied to the fetal dataset that was reconstructed with offline STCR approach. Then, an increasing number of dynamic modes (shown as percentage of maximum available modes) were used to generate a DMD reconstructed image series. The model appropriateness is defined as the RMSE normalized by the input energy, given a DMD reconstruction. Note that as the number of modes increases, the normalized RMSE decreases; however, it is always nonzero as the DMD is not an orthogonal transformation. We show an example from a healthy pregnancy (34w 6d, maternal BMI 24) with a high model error. As seen from the video and the line profiles, DMD can represent the original input dynamics faithfully.

**Video S3:** Impact of residue scale factor (α) on de‐aliasing performance for fetal cardiac RT‐MRI, movie of Figure 4. The residual signal, difference between the gridded input image series and the DMD model estimation, can be used to further suppress the aliasing. Specifically, the residual signal is scaled with parameter α∈[0,1] (residue scale factor) and added to the estimated aliasing with little computational overhead (no iterations). The effect of α is shown here for 0, 0.5 and 0.8 on an example fetal dataset with bulk motion (marked in the line profile). Larger α values support better dealiasing performance at the cost of temporal smoothing, while smaller α values preserve the dynamics better at the cost of increased aliasing. We have selected α=0.5 as it provides a good qualitative balance between the de‐aliasing performance and preserving the dynamics.

**Video S4:** De‐aliasing performance in a healthy adult volunteer (M, 49 years old, BMI 29), movie of Figure 6. DMD based dealiazing is applied to the gridded image series; results are shown together with STCR results at the same temporal resolution.

**Video S5:** De‐aliasing performance in an adult volunteer experiencing premature ventricular contractions during the scan. Results are shown with gridding and STCR at two temporal resolutions (4 and 8 TR/frame). The irregular beat is highlighted in the line intensity profiles. The proposed approach can perform de‐aliasing while preserving the underlying motion, in this case, an elongated irregular heartbeat.

**Video S6:** Feasibility of DMD for low‐latency fetal cardiac RT‐MRI, movie of Figure 7. Gridding, Gridding + DMD based de‐aliasing and STCR results are shown on a dataset from a healthy pregnancy (maternal age/BMI: 32/24, gestation age 34 weeks 6 days). The proposed reconstruction can perform rapid de‐aliasing to localize the fetal heart with a good temporal resolution, which can be useful to guide localization.

**Video S7:** Feasibility of DMD for low‐latency fetal cardiac RT‐MRI, in a subject with high maternal BMI. Gridding, Gridding + DMD based de‐aliasing and STCR results are shown on a dataset from a healthy pregnancy (maternal age/BMI: 35/47, gestation age 31 week 4 days). The fetal heart (annotated) is visible, and heart motion is preserved after DMD based de‐aliasing.

## Data Availability

Sample reconstruction code is available on GitHub: https://github.com/usc‐mrel/DMD‐RT. A snapshot of the reconstruction code and example data are available via Zenodo: https://zenodo.org/records/17025309 and https://zenodo.org/records/17014548.

## References

[1] P. S. Rajiah , C. J. François , and T. Leiner , “Cardiac MRI: State of the Art,” Radiology 307, no. 3 (2023): e223008, 10.1148/radiol.223008. 37039684

[2] C. M. Kramer , J. Barkhausen , C. Bucciarelli‐Ducci , S. D. Flamm , R. J. Kim , and E. Nagel , “Standardized Cardiovascular Magnetic Resonance Imaging (CMR) Protocols: 2020 Update,” Journal of Cardiovascular Magnetic Resonance 22, no. 1 (2020): 17, 10.1186/s12968-020-00607-1. 32089132 PMC7038611

[3] N. Nita , J. Kersten , A. Pott , et al., “Real‐Time Spiral CMR Is Superior to Conventional Segmented Cine‐Imaging for Left‐Ventricular Functional Assessment in Patients With Arrhythmia,” Journal of Clinical Medicine 11, no. 8 (2022): 2088, 10.3390/jcm11082088. 35456181 PMC9025940

[4] M. M. Masaracchia , M. J. Tsapakos , N. J. McNulty , and M. L. Beach , “Changing the Paradigm for Diagnostic MRI in Pediatrics: Don't Hold Your Breath,” Pediatric Anesthesia 27, no. 9 (2017): 880–884, 10.1111/pan.13165. 28504359

[5] T. M. Vollbrecht , M. M. Bissell , F. Kording , et al., “Fetal Cardiac MRI Using Doppler US Gating: Emerging Technology and Clinical Implications,” Radiology: Cardiothoracic Imaging 6, no. 2 (2024): e230182, 10.1148/ryct.230182. 38602469 PMC11056758

[6] F. Kording , J. Yamamura , M. T. De Sousa , et al., “Dynamic Fetal Cardiovascular Magnetic Resonance Imaging Using Doppler Ultrasound Gating,” Journal of Cardiovascular Magnetic Resonance 20, no. 1 (2018): 17, 10.1186/s12968-018-0440-4. 29530064 PMC5846256

[7] M. B. L. Falcão , L. Di Sopra , L. Ma , et al., “Pilot Tone Navigation for Respiratory and Cardiac Motion‐Resolved Free‐Running 5D Flow MRI,” Magnetic Resonance in Medicine 87, no. 2 (2022): 718–732, 10.1002/mrm.29023. 34611923 PMC8627452

[8] P. Speier and M. Bacher , “Skip the Electrodes, But Not A Beat: The Engineering Behind the Beat Sensor,” Magnetom Flash 84 (2023): 106–113.

[9] B. Tasdelen , E. Yagiz , B. R. Cinbis , Y. Tian , and K. S. Nayak , “Contactless Cardiac Gating at 0.55T Using High‐Amplitude Pilot Tone With Interference Cancellation (HAPTIC),” Magnetic Resonance in Medicine 94, no. 3 (2025): 1182–1190, 10.1002/mrm.30528. 40228074 PMC12202719

[10] “Performance of Simultaneous Cardiac‐Respiratory Self‐Gated Three‐Dimensional MR Imaging of the Heart: Initial Experience,” 10.1148/radiol.10091103. 20501728

[11] A. C. Larson , R. D. White , G. Laub , E. R. McVeigh , D. Li , and O. P. Simonetti , “Self‐Gated Cardiac Cine MRI,” Magnetic Resonance in Medicine 51, no. 1 (2004): 93–102, 10.1002/mrm.10664. 14705049 PMC2396326

[12] I. Montón Quesada , A. C. Ogier , M. Ishida , et al., “Self‐Gated Free‐Running 5D Whole‐Heart MRI Using Blind Source Separation for Automated Cardiac Motion Extraction,” Magnetic Resonance in Medicine 93, no. 3 (2025): 961–974, 10.1002/mrm.30322. 39385391 PMC11680725

[13] K. S. Nayak , “Response to Letter to the Editor: “Nomenclature for Real‐Time Magnetic Resonance Imaging.”,” Magnetic Resonance in Medicine 82, no. 2 (2019): 525–526, 10.1002/mrm.27770. 31025418

[14] K. S. Nayak , Y. Lim , A. E. Campbell‐Washburn , and J. Steeden , “Real‐Time Magnetic Resonance Imaging,” Journal of Magnetic Resonance Imaging (JMRI) 55, no. 1 (2022): 81–99, 10.1002/jmri.27411. 33295674 PMC8435094

[15] C. S. De Jonge , R. M. Gollifer , A. J. Nederveen , et al., “Dynamic MRI for Bowel Motility Imaging–How Fast and How Long?,” British Journal of Radiology 91 (2018): 20170845, 10.1259/bjr.20170845. 29474115 PMC6209475

[16] Y. Tian and K. S. Nayak , “Real‐Time Water/Fat Imaging at 0.55T With Spiral Out‐In‐Out‐In Sampling,” Magnetic Resonance in Medicine 91, no. 2 (2024): 649–659, 10.1002/mrm.29885. 37815020 PMC10841523

[17] C. B. Shaw , B. H. Foster , M. Borgese , et al., “Real‐Time Three‐Dimensional MRI for the Assessment of Dynamic Carpal Instability,” PLoS One 14, no. 9 (2019): e0222704, 10.1371/journal.pone.0222704. 31536561 PMC6752861

[18] Z. Wu , W. Chen , M. C. K. Khoo , S. L. Davidson Ward , and K. S. Nayak , “Evaluation of Upper Airway Collapsibility Using Real‐Time MRI: Real‐Time MRI for Sleep Apnea,” Journal of Magnetic Resonance Imaging 44, no. 1 (2016): 158–167, 10.1002/jmri.25133. 26708099 PMC6768084

[19] B. M. Kozak , C. Jaimes , J. Kirsch , and M. S. Gee , “MRI Techniques to Decrease Imaging Times in Children,” Radiographics 40, no. 2 (2020): 485–502, 10.1148/rg.2020190112. 32031912

[20] A. E. Campbell‐Washburn , M. A. Tavallaei , M. Pop , et al., “Real‐Time MRI Guidance of Cardiac Interventions,” Journal of Magnetic Resonance Imaging 46, no. 4 (2017): 935–950, 10.1002/jmri.25749. 28493526 PMC5675556

[21] S. Winkelmann , T. Schaeffter , T. Koehler , H. Eggers , and O. Doessel , “An Optimal Radial Profile Order Based on the Golden Ratio for Time‐Resolved MRI,” IEEE Transactions on Medical Imaging 26, no. 1 (2007): 68–76, 10.1109/TMI.2006.885337. 17243585

[22] S. J. Riederer , T. Tasciyan , F. Farzaneh , J. N. Lee , R. C. Wright , and R. J. Herfkens , “MR Fluoroscopy: Technical Feasibility,” Magnetic Resonance in Medicine 8, no. 1 (1988): 1–15, 10.1002/mrm.1910080102. 3173063

[23] P. Kellman , J. M. Sorger , F. H. Epstein , and E. R. McVeigh , “Low Latency Temporal Filter Design for Real‐Time MRI Using UNFOLD,” Magnetic Resonance in Medicine 44, no. 6 (2000): 933–939, 10.1002/1522-2594(200012)44:6<933::AID-MRM15>3.0.CO;2-I. 11108631 PMC2169202

[24] U. Sumbul , J. M. Santos , and J. M. Pauly , “A Practical Acceleration Algorithm for Real‐Time Imaging,” IEEE Transactions on Medical Imaging 28, no. 12 (2009): 2042–2051, 10.1109/tmi.2009.2030474. 19709964 PMC3155727

[25] D. H. Le , P. Kumar , E. Yagiz , Y. Tian , and K. S. Nayak , “Online Spatiotemporally Constrained Reconstruction for Real‐Time Interactive MRI,” Magnetic Resonance in Medicine 95, no. 3 (2026): 1644–1652, 10.1002/mrm.70131. 41108664 PMC12746365

[26] M. Uecker , S. Zhang , and J. Frahm , “Nonlinear Inverse Reconstruction for Real‐Time MRI of the Human Heart Using Undersampled Radial FLASH,” Magnetic Resonance in Medicine 63, no. 6 (2010): 1456–1462, 10.1002/mrm.22453. 20512847

[27] Z. He , Y. N. Zhu , S. Qiu , et al., “Low‐Rank and Framelet Based Sparsity Decomposition for Interventional MRI Reconstruction,” IEEE Transactions on Biomedical Engineering 69, no. 7 (2022): 2294–2304, 10.1109/TBME.2022.3142129. 35015631

[28] O. Jaubert , J. Montalt‐Tordera , D. Knight , S. Arridge , J. Steeden , and V. Muthurangu , “HyperSLICE: HyperBand Optimized Spiral for Low‐Latency Interactive Cardiac Examination,” Magnetic Resonance in Medicine 91, no. 1 (2024): 266–279, 10.1002/mrm.29855. 37799087 PMC10953456

[29] S. Siddiq , V. Murray , N. Tyagi , et al., “MR Signature Matching (MRSIGMA) Implementation for True Real‐Time Free‐Breathing Volumetric Imaging With Sub‐200 ms Latency on an MR‐Linac,” Magnetic Resonance in Medicine 92, no. 3 (2024): 1162–1176, 10.1002/mrm.30097. 38576131 PMC11209806

[30] D. E. J. Waddington , N. Hindley , N. Koonjoo , et al., “Real‐Time Radial Reconstruction With Domain Transform Manifold Learning for MRI‐Guided Radiotherapy,” Medical Physics 50, no. 4 (2023): 1962–1974, 10.1002/mp.16224. 36646444 PMC10809819

[31] G. Yang , S. Yu , H. Dong , et al., “DAGAN: Deep De‐Aliasing Generative Adversarial Networks for Fast Compressed Sensing MRI Reconstruction,” IEEE Transactions on Medical Imaging 37, no. 6 (2018): 1310–1321, 10.1109/TMI.2017.2785879. 29870361

[32] O. Jaubert , J. Montalt‐Tordera , D. Knight , et al., “Real‐Time Deep Artifact Suppression Using Recurrent U‐Nets for Low‐Latency Cardiac MRI,” Magnetic Resonance in Medicine 86, no. 4 (2021): 1904–1916, 10.1002/mrm.28834. 34032308 PMC8613539

[33] M. A. Morales , S. Assana , X. Cai , et al., “An Inline Deep Learning Based Free‐Breathing ECG‐Free Cine for Exercise Cardiovascular Magnetic Resonance,” Journal of Cardiovascular Magnetic Resonance 24, no. 1 (2022): 47, 10.1186/s12968-022-00879-9. 35948936 PMC9367083

[34] L. Fan , D. Shen , H. Haji‐Valizadeh , et al., “Rapid Dealiasing of Undersampled, Non‐Cartesian Cardiac Perfusion Images Using U‐Net,” NMR in Biomedicine 33, no. 5 (2020): e4239, 10.1002/nbm.4239. 31943431 PMC7165063

[35] B. Pialot , F. Guidi , P. Muleki‐Seya , E. Boni , A. Ramalli , and F. Varray , “Dynamic Mode Decomposition as a Framework for Denoising Ultrafast Power Doppler Images,” Computer Methods and Programs in Biomedicine 276 (2026): 109221, 10.1016/j.cmpb.2025.109221. 41475187

[36] J. M. Kunert‐Graf , K. M. Eschenburg , D. J. Galas , J. N. Kutz , S. D. Rane , and B. W. Brunton , “Extracting Reproducible Time‐Resolved Resting State Networks Using Dynamic Mode Decomposition,” Frontiers in Computational Neuroscience 13 (2019): 75, 10.3389/fncom.2019.00075. 31736734 PMC6834549

[37] J. N. Kutz , X. Fu , and S. L. Brunton , “Multiresolution Dynamic Mode Decomposition,” SIAM Journal on Applied Dynamical Systems 15, no. 2 (2016): 713–735, 10.1137/15m1023543.

[38] B. Kramer , P. Grover , P. Boufounos , S. Nabi , and M. Benosman , “Sparse Sensing and DMD‐Based Identification of Flow Regimes and Bifurcations in Complex Flows,” SIAM Journal on Applied Dynamical Systems 16, no. 2 (2017): 1164–1196, 10.1137/15m104565x.

[39] S. Ikeda , K. Kawano , S. Watanabe , O. Yamashita , and Y. Kawahara , “Predicting Behavior Through Dynamic Modes in Resting‐State fMRI Data,” NeuroImage 247 (2022): 118801, 10.1016/j.neuroimage.2021.118801. 34896588

[40] E. Doyle , E. Yagiz , S. Cui , J. Detterich , R. Kato , and K. Nayak , “Lung Ventilation‐Perfusion Imaging Approaches in the Single Ventricle Heart,” (2025), 10.58530/2025/0912.

[41] E. Ilicak , S. Ozdemir , J. Zapp , L. R. Schad , and F. G. Zöllner , “Dynamic Mode Decomposition of Dynamic MRI for Assessment of Pulmonary Ventilation and Perfusion,” Magnetic Resonance in Medicine 90, no. 2 (2023): 761–769, 10.1002/mrm.29656. 36989180

[42] P. J. Schmid , “Dynamic Mode Decomposition of Numerical and Experimental Data,” Journal of Fluid Mechanics 656 (2010): 5–28, 10.1017/S0022112010001217.

[43] J. N. Kuntz , S. L. Brunton , B. W. Brunton , and J. L. Proctor , Dynamic Mode Decomposition: Data Driven Modeling of Complex Systems (Society for Industrial and Applied Mathematics, 2016), 10.1137/1.9781611974508.

[44] J. H. Tu , C. W. Rowley , D. M. Luchtenburg , S. L. Brunton , and J. N. Kutz , “On Dynamic Mode Decomposition: Theory and Applications,” Journal of Computational Dynamics 1, no. 2 (2014): 391–421, 10.3934/jcd.2014.1.391.

[45] A. E. Campbell‐Washburn , R. Ramasawmy , M. C. Restivo , et al., “Opportunities in Interventional and Diagnostic Imaging by Using High‐Performance Low‐Field‐Strength MRI,” Radiology 293, no. 2 (2019): 384–393, 10.1148/radiol.2019190452. 31573398 PMC6823617

[46] S. Ponrartana , H. N. Nguyen , S. X. Cui , et al., “Low‐Field 0.55 T MRI Evaluation of the Fetus,” Pediatric Radiology 53, no. 7 (2023): 1469–1475, 10.1007/s00247-023-05604-x. 36882594 PMC10276075

[47] Y. Tian , J. Detterich , J. D. Pruetz , E. Yagiz , J. C. Wood , and K. S. Nayak , “Feasibility of Fetal Cardiac Function and Anatomy Assessment by Real‐Time Spiral Balanced Steady‐State Free Precession Magnetic Resonance Imaging at 0.55T,” Journal of Cardiovascular Magnetic Resonance 27, no. 1 (2025): 101130, 10.1016/j.jocmr.2024.101130. 39638149 PMC12182825

[48] J. M. Santos , G. A. Wright , and J. M. Pauly , “Flexible Real‐Time Magnetic Resonance Imaging Framework,” in The 26th Annual International Conference of the IEEE Engineering in Medicine and Biology Society, vol. 3 (IEEE, 2004), 1048–1051, 10.1109/IEMBS.2004.1403343. 17271862

[49] B. Tasdelen and P. Kumar , “2D/3D Spiral Sequences With PyPulseq,” accessed April 25, 2025, github.com/usc‐mrel/rtspiral_pypulseq.

[50] K. S. Ravi , S. Geethanath , and T. J. Vaughan , “PyPulseq: A Python Package for MRI Pulse Sequence Design,” Journal of Open Source Software 4, no. 42 (2019): 1725, 10.21105/joss.01725.

[51] K. S. Nayak , B. A. Hargreaves , B. S. Hu , D. G. Nishimura , J. M. Pauly , and C. H. Meyer , “Spiral Balanced Steady‐State Free Precession Cardiac Imaging,” Magnetic Resonance in Medicine 53, no. 6 (2005): 1468–1473, 10.1002/mrm.20489. 15906302

[52] A. E. Campbell‐Washburn , H. Xue , R. J. Lederman , A. Z. Faranesh , and M. S. Hansen , “Real‐Time Distortion Correction of Spiral and Echo Planar Images Using the Gradient System Impulse Response Function,” Magnetic Resonance in Medicine 75, no. 6 (2016): 2278–2285, 10.1002/mrm.25788. 26114951 PMC4691439

[53] Y. Tian , S. X. Cui , Y. Lim , N. G. Lee , Z. Zhao , and K. S. Nayak , “Contrast‐Optimal Simultaneous Multi‐Slice bSSFP Cine Cardiac Imaging at 0.55 T,” Magnetic Resonance in Medicine 89, no. 2 (2023): 746–755, 10.1002/mrm.29472. 36198043 PMC9712243

[54] E. Yagiz , P. Garg , S. Y. Cen , K. S. Nayak , and Y. Tian , “Simultaneous Multi‐Slice Cardiac Real‐Time MRI at 0.55T,” Magnetic Resonance in Medicine 93, no. 4 (2025): 1723–1732, 10.1002/mrm.30364. 39506513 PMC11782716

[55] Y. Tian , J. Mendes , A. Pedgaonkar , et al., “Feasibility of Multiple‐View Myocardial Perfusion MRI Using Radial Simultaneous Multi‐Slice Acquisitions,” PLoS One 14, no. 2 (2019): e0211738, 10.1371/journal.pone.0211738. 30742641 PMC6370206

[56] Z. Zhao , Y. Lim , D. Byrd , S. Narayanan , and K. S. Nayak , “Improved 3D Real‐Time MRI of Speech Production,” Magnetic Resonance in Medicine 85, no. 6 (2021): 3182–3195, 10.1002/mrm.28651. 33452722

[57] Y. Tian , J. Mendes , B. Wilson , et al., “Whole‐Heart, Ungated, Free‐Breathing, Cardiac‐Phase‐Resolved Myocardial Perfusion MRI by Using Continuous Radial Interleaved Simultaneous Multi‐Slice Acquisitions at sPoiled Steady‐State (CRIMP),” Magnetic Resonance in Medicine 84, no. 6 (2020): 3071–3087, 10.1002/mrm.28337. 32492235 PMC7710603

[58] D. O. Walsh , A. F. Gmitro , and M. W. Marcellin , “Adaptive Reconstruction of Phased Array MR Imagery,” Magnetic Resonance in Medicine 43, no. 5 (2000): 682–690, 10.1002/(SICI)1522-2594(200005)43:5<682::AID-MRM10>3.0.CO;2-G. 10800033

[59] M. R. Jovanović , P. J. Schmid , and J. W. Nichols , “Sparsity‐Promoting Dynamic Mode Decomposition,” Physics of Fluids 26, no. 2 (2014): 024103, 10.1063/1.4863670.

[60] H. Von Kleist , M. Buehrer , S. Kozerke , et al., “Cardiac Self‐Gating Using Blind Source Separation for 2D Cine Cardiovascular Magnetic Resonance Imaging,” Magnetic Resonance Imaging 81 (2021): 42–52, 10.1016/j.mri.2021.04.008. 33905835

[61] S. Pildner Von Steinburg , A. L. Boulesteix , C. Lederer , et al., “What Is the “Normal” Fetal Heart Rate?,” PeerJ 1 (2013): e82, 10.7717/peerj.82. 23761161 PMC3678114

[62] R. Zhang , P. Isola , A. A. Efros , E. Shechtman , and O. Wang , “The Unreasonable Effectiveness of Deep Features as a Perceptual Metric,” preprint, arXiv, 2018, 1801.03924, 10.48550/arXiv.1801.03924.

[63] S. Ravishankar and Y. Bresler , “MR Image Reconstruction From Highly Undersampled k‐Space Data by Dictionary Learning,” IEEE Transactions on Medical Imaging 30, no. 5 (2011): 1028–1041, 10.1109/TMI.2010.2090538. 21047708

[64] Z. Wang , A. C. Bovik , H. R. Sheikh , and E. P. Simoncelli , “Image Quality Assessment: From Error Visibility to Structural Similarity,” IEEE Transactions on Image Processing 13, no. 4 (2004): 600–612, 10.1109/TIP.2003.819861. 15376593

[65] R. Okuta , Y. Unno , D. Nishino , S. Hido , and C. Loomis , “CuPy: A NumPy‐Compatible Library for NVIDIA GPU Calculations,” 2017.

[66] M. C. Restivo , R. Ramasawmy , W. P. Bandettini , D. A. Herzka , and A. E. Campbell‐Washburn , “Efficient Spiral In‐Out and EPI Balanced Steady‐State Free Precession Cine Imaging Using a High‐Performance 0.55T MRI,” Magnetic Resonance in Medicine 84, no. 5 (2020): 2364–2375, 10.1002/mrm.28278. 32291845 PMC7402011

[67] T. Rogers , K. Ratnayaka , J. M. Khan , et al., “CMR Fluoroscopy Right Heart Catheterization for Cardiac Output and Pulmonary Vascular Resistance: Results in 102 Patients,” Journal of Cardiovascular Magnetic Resonance 19, no. 1 (2016): 54, 10.1186/s12968-017-0366-2. PMC5530573 28750642

[68] K. Keijnemans , P. T. S. Borman , B. W. Raaymakers , and M. F. Fast , “Effectiveness of Visual Biofeedback–Guided Respiratory‐Correlated 4D‐MRI for Radiotherapy Guidance on the MR‐Linac,” Magnetic Resonance in Medicine 91, no. 1 (2024): 297–311, 10.1002/mrm.29857. 37799101

[69] D. T. To , J. P. Kim , R. G. Price , I. J. Chetty , and C. K. Glide‐Hurst , “Impact of Incorporating Visual Biofeedback in 4D MRI,” Journal of Applied Clinical Medical Physics 17, no. 3 (2016): 128–137, 10.1120/jacmp.v17i3.6017. PMC5690930 27167270

