# Supplementary material for: Dynamic Mode Decomposition (DMD) for Low‐Latency Real‐Time Cardiac MRI
Source: Magn Reson Med. 2026 Mar 30;96(2):623–34. doi: 10.1002/mrm.70360 (PMC13269186; doi:10.1002/mrm.70360)
Supplement: Supplementary file 1 — Figure S1: Impact of DMD buffer length on image quality metrics, for fetal cardiac RT‐MRI. We performed gridding on all fetal datasets. Then retrospectively, we applied the proposed low‐latency reconstruction using different buffer lengths. The performance of the de‐aliasing was assessed using spatiotemporally constrained reconstruction (STCR) as a reference by normalized RMSE (NRMSE), high‐frequency error norm (HFEN), learned perceptual image patch similarity (LPIPS), peak signal‐to‐noise ratio (PSNR), and structural similarity index measure (SSIM). We found the sliding window width of 848 ms (Nf= 20 frames at 8 TR/frame and Nf= 40 frames at 4 TR/frame) to be optimal. Table S1: Summary of image quality metrics for the adult and fetal cohort at two temporal resolutions (4 and 8 TR/frame). The performance of the de‐aliasing was assessed using STCR reconstructions by normalized RMSE (NRMSE), high‐frequency error norm (HFEN), learned perceptual image patch similarity (LPIPS), peak signal‐to‐noise ratio (PSNR), and structural similarity index measure (SSIM). Arrows indicate the preferred direction in a given metric (higher ↑/lower ↓). The same image quality metrics were obtained for gridded images as a baseline. The proposed DMD‐based approach (gridding + DMD) shows improvement of the image quality providing low‐latency reconstruction (can provide framerates of 21 ms). [file MRM-96-623-s002.docx]

**Supplemental Material Captions**


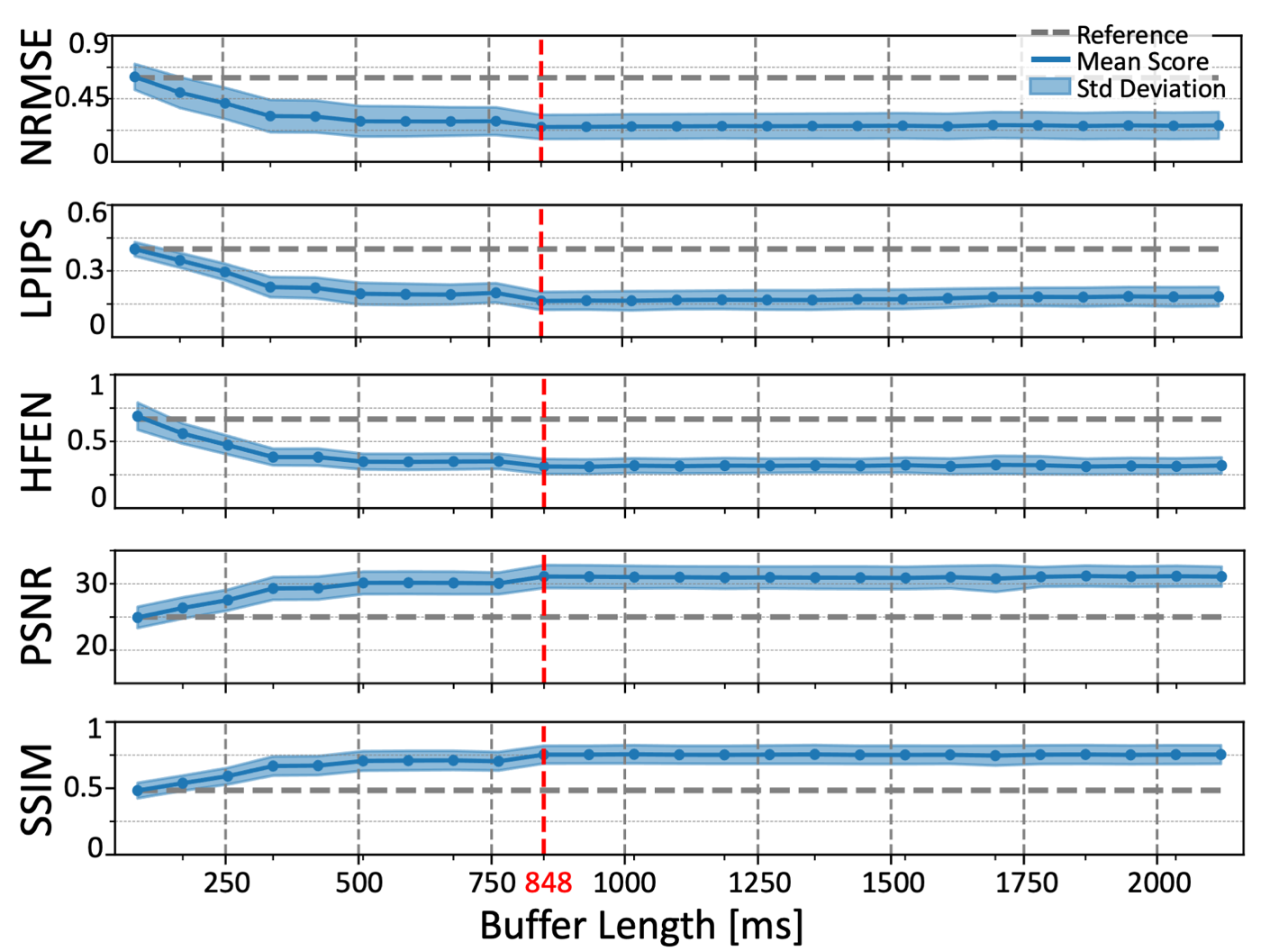


**Figure S1**: **Impact of DMD buffer length on image quality metrics, for fetal cardiac RT-MRI.** We performed gridding on all fetal datasets. Then retrospectively, we applied the proposed low-latency reconstruction using different buffer lengths. The performance of the de-aliasing was assessed using spatiotemporally constrained reconstruction (**STCR**) as a reference by normalized RMSE (**NRMSE**), high-frequency error norm (**HFEN**), learned perceptual image patch similarity (**LPIPS**), peak signal-to-noise ratio (**PSNR**) and structural similarity index measure (**SSIM**). We found the sliding window width of 848 ms ($N_{f}=$ 20 frames at 8 TR/frame and $N_{f}=$ 40 frames at 4 TR/frame) to be optimal.

|  |  | NRMSE ($\boldsymbol{\downarrow}$) | LPIPS ($\boldsymbol{\downarrow}$) | HFEN ($\boldsymbol{\downarrow}$) | PSNR($\boldsymbol{\uparrow}$) | SSIM ($\boldsymbol{\uparrow}$) |
| --- | --- | --- | --- | --- | --- | --- |
| 4 TR/frame | Adult, DMD | 0.29 $\pm$ 0.16 | 0.13 $\pm$ 0.07 | 0.27 $\pm$ 0.11 | 34.35 $\pm$ 3.89 | 0.67 $\pm$ 0.11 |
|  | Adult, Grid | 0.66 $\pm$ 0.05 | 0.34 $\pm$ 0.05 | 0.64 $\pm$ 0.03 | 26.07 $\pm$ 2.63 | 0.43 $\pm$ 0.10 |
|  | Fetal, DMD | 0.29 $\pm$ 0.08 | 0.19 $\pm$ 0.04 | 0.30 $\pm$ 0.05 | 33.18 $\pm$ 2.77 | 0.69 $\pm$ 0.07 |
|  | Fetal, Grid | 0.69 $\pm$ 0.08 | 0.39 $\pm$ 0.04 | 0.65 $\pm$ 0.10 | 25.56 $\pm$ 2.82 | 0.48 $\pm$ 0.08 |
| 8 TR/frame | Adult, DMD | 0.19 $\pm$ 0.07 | 0.08 $\pm$ 0.05 | 0.20 $\pm$ 0.03 | 37.18 $\pm$ 3.44 | 0.76 $\pm$ 0.10 |
|  | Adult, Grid | 0.49 $\pm$ 0.07 | 0.27 $\pm$ 0.05 | 0.47 $\pm$ 0.04 | 28.62 $\pm$ 2.52 | 0.50 $\pm$0.07 |
|  | Fetal, DMD | 0.24 $\pm$ 0.09 | 0.17 $\pm$ 0.04 | 0.26 $\pm$ 0.04 | 33.88 $\pm$ 2.97 | 0.72 $\pm$ 0.07 |
|  | Fetal, Grid | 0.55 $\pm$ 0.08 | 0.41 $\pm$ 0.02 | 0.53 $\pm$ 0.08 | 26.41 $\pm$ 2.95 | 0.46 $\pm$ 0.07 |

**Table S1: Summary of image quality metrics for the adult and fetal cohort at two temporal resolutions (4 TR/frame and 8 TR/frame).** The performance of the de-aliasing was assessed using STCR reconstructions by normalized RMSE (**NRMSE**), high-frequency error norm (**HFEN**), learned perceptual image patch similarity (**LPIPS**), peak signal-to-noise ratio (**PSNR**) and structural similarity index measure (**SSIM**). Arrows indicate the preferred direction in a given metric (higher ↑ / lower ↓). The same image quality metrics were obtained for gridded images as a baseline. The proposed DMD-based approach (gridded + DMD) shows improvement of the image quality providing low-latency reconstruction (can provide framerates of 21 ms).

**Video S1: Representative example of DMD model capacity for adult cardiac RT-MRI.** DMD was applied to the spatiotemporally constrained reconstruction (**STCR**) results. Then, a varying number of dynamic modes (shown as percentage of maximum available modes) were used to generate a DMD reconstructed image series. The model appropriateness is defined as the root-mean-square error (**RMSE**) normalized by the input energy, given a DMD reconstruction. **Figure 2A** shows the normalized RMSE of model error with respect to the number of modes on all adult datasets. Here, an example dataset (M; 49 yo; BMI 29) with one of the highest normalized RMSE is shown (movie of **Figure 2B**). As can be seen from the DMD representations at 50% and 100%, qualitatively DMD can represent the original input dynamics faithfully. **Video S2** shows the same analysis for the fetal dataset.

**Video S2:** **DMD model appropriateness for 2D fetal cardiac RT-MRI.** DMD was applied to the fetal dataset that was reconstructed with offline **STCR** approach. Then, an increasing number of dynamic modes (shown as percentage of maximum available modes) were used to generate a DMD reconstructed image series. The model appropriateness is defined as the RMSE normalized by the input energy, given a DMD reconstruction. Note that as the number of modes increases, the normalized RMSE decreases; however, it is always non-zero as the DMD is not an orthogonal transformation. We show an example from a healthy pregnancy (34w 6d, maternal BMI 24) with a high model error. As seen from the video and the line profiles, DMD can represent the original input dynamics faithfully.

**Video S3:** **Impact of residue scale factor (**$\boldsymbol{\alpha}$**) on de-aliasing performance for fetal cardiac RT-MRI**, **movie of Figure 4**. The residual signal, difference between the gridded input image series and the DMD model estimation, can be used to further suppress the aliasing. Specifically, the residual signal is scaled with parameter $\alpha\in[0,1]$ (residue scale factor) and added to the estimated aliasing with little computational overhead (no iterations). The effect of $\alpha$ is shown here for 0, 0.5 and 0.8 on an example fetal dataset with bulk motion (marked in the line profile). Larger $\alpha$ values support better dealiasing performance at the cost of temporal smoothing, while smaller $\alpha$ values preserve the dynamics better at the cost of increased aliasing. We have selected $\alpha=0.5$ as it provides a good qualitative balance between the de-aliasing performance and preserving the dynamics.

**Video S4: De-aliasing performance in a healthy adult volunteer (M, 49 yo, BMI 29), movie of Figure 6.** DMD based dealiasing is applied to the gridded image series; results are shown together with STCR results at the same temporal resolution.

**Video S5:** **De-aliasing performance in an adult volunteer experiencing premature ventricular contractions during the scan**. Results are shown with gridding and STCR at two temporal resolutions (4 TR/frame and 8 TR/frame). The irregular beat is highlighted in the line intensity profiles. The proposed approach can perform de-aliasing while preserving the underlying motion, in this case, an elongated irregular heartbeat.

**Video S6:** **Feasibility of DMD for low-latency fetal cardiac RT-MRI, movie of Figure 7.** Gridding, Gridding + DMD based de-aliasing and STCR results are shown on a dataset from a healthy pregnancy (maternal age/BMI: 32/24, gestation age 34w 6d). The proposed reconstruction can perform rapid de-aliasing to localize the fetal heart with a good temporal resolution, which can be useful to guide localization.

**Video S7:** **Feasibility of DMD for low-latency fetal cardiac RT-MRI, in a subject with high maternal BMI.** Gridding, Gridding + DMD based de-aliasing and STCR results are shown on a dataset from a healthy pregnancy (maternal age/BMI: 35/47, gestation age 31w 4d). The fetal heart (annotated) is visible, and heart motion is preserved after DMD based de-aliasing.
